# Optical coherence tomography measurements in Huntington’s disease: a systematic review and meta-analysis

**DOI:** 10.1007/s00415-024-12634-4

**Published:** 2024-08-26

**Authors:** Mahdi Gouravani, Sepehr Fekrazad, Asma Mafhoumi, Moein Ashouri, Delia Cabrera DeBuc

**Affiliations:** 1 School of Medicine, Tehran University of Medical Sciences, Tehran, Iran; 2 Miller School of Medicine, Bascom Palmer Eye Institute, University of Miami, 900 NW 17 Street, Miami, FL 33136 USA; 3 International Network for Photomedicine and Photodynamic Therapy (INPMPDT), Universal Scientific Education and Research Network (USERN), Tehran, Iran

**Keywords:** Optical coherence tomography, Retina, Choroid, Huntington's disease, Meta-analysis

## Abstract

**Background:**

A connection has been established between ocular structural changes and various neurodegenerative diseases. Several studies utilizing optical coherence tomography (OCT) have detected signs of ocular structural alterations among individuals with Huntington's disease (HD). The inconsistent results reported in the literature regarding alterations in the retina and choroid encouraged us to conduct this systematic review and meta-analysis to accumulate the findings.

**Methods:**

A systematic search was carried out in three electronic databases (PubMed, Embase, Scopus) to find studies reporting OCT measurements in HD cases compared with healthy controls (HC). A fixed-effects or random-effects meta-analysis was conducted according to the detected heterogeneity level. Furthermore, subgroup and sensitivity analyses, meta-regression, and quality assessment were performed.

**Results:**

Eleven studies were included in the systematic review and 9 studies with a total population of 452 participants (241 cases, and 211 HC) underwent meta-analysis. Results of the analysis denoted that subfoveal choroid had a significantly reduced thickness in HD eyes compared to HC (*p* < 0.0001). Moreover, our analysis indicated that HD cases had a significantly thinner average (*p* = 0.0130) and temporal peripapillary retinal nerve fiber layer (pRNFL) (*p* = 0.0012) than HC. However, subjects with pre-HD had insignificant differences in average (*p* = 0.44) and temporal pRNFL thickness (*p* = 0.33) with the HC group.

**Conclusion:**

Results of the current systematic review and meta-analysis revealed the significant thinning of average and temporal pRNFL and subfoveal choroid in HD compared to HC. However, OCT currently might be considered insensitive to be applied in the pre-HD population at least until further longitudinal investigations considering variables such as the duration between OCT measurement and disease onset validating OCT as a routine diagnostic tool in HD clinics.

**Supplementary Information:**

The online version contains supplementary material available at 10.1007/s00415-024-12634-4.

## Introduction

Huntington's disease (HD) is a fatal, autosomal dominant neurodegenerative disorder caused by CAG trinucleotide expansion repeat (usually over 40 repeats) within exon 1 of the huntingtin (HTT) gene at the fourth chromosome encoding the HTT protein [1–4]. The worldwide prevalence of HD is 2.7 per 100,000, and the incidence is reported as 0.38 per 100,000. However, there is more than ten-fold variation in HD prevalence across different geographical areas [5]. Clinical symptoms of HD are characterized by a progressive disturbance in motor, cognitive, and psychiatric functions, manifested typically between the 2nd and 4th decades of life, although they can occur earlier or later [1, 4]. Individuals carrying HD genes without symptoms are considered pre-manifest (pre-HD), while those with both genetic and clinical evidence are classified as manifest HD [6].

HD predominantly affects the striatum (caudate nucleus and putamen) and cerebral cortex in a posterior-to-anterior pattern involving the visual cortex [2, 7]. Evidence supports that neuronal dysfunction in HD can extend beyond the brain structures and affect the retinal layers, which have the same origin as the brain [4, 7]. The literature also indicates that the retina contains HTT protein. The presence of dysmorphic HTT protein in the retina in HD can give rise to functional or structural impairment of the visual pathways [1]. Progressive neurodegeneration in HD also affects photoreceptors and neurotransmitters of the retinal layers, resulting in impaired neurotransmission to cortical ribbon and visual dysfunction [7, 8]. Patients with pre-manifest HD may experience ocular symptoms, such as increased blinking, slower horizontal eye movement, slower saccade speed, and longer saccade latency, all thought to be related to HD [3]. Animal model studies revealed loss of rod and cone function, photoreceptor degeneration, insufficiency in cone response to electroretinogram, as well as visual impairment, retinal dystrophy in transgenic mice, and photoreceptor degeneration in drosophila [9, 10]. Although long-term clinical observations demonstrated that HD patients may experience no visual complication, several studies have shown changes in retinal structures and visuospatial, visuomotor, and visual memory function [1, 7, 8, 10–12].

Optical coherence tomography (OCT) is a novel, non-invasive, light-based imaging technique with rapid data acquisition time, which provides micrometer-scale resolution, cross-sectional, three-dimensional images of posterior structures of the eye, including retina and choroid [4, 10, 13]. Advancements in OCT imaging techniques from earlier time-domain OCT (TD-OCT) to recent versions of spectral-domain (SD-OCT) and swept-source OCT (SS-OCT) have improved image resolutions [14]. In neurodegenerative diseases, including multiple sclerosis (MS), Alzheimer’s disease (AD), and Parkinson’s disease (PD), OCT metrics such as retinal nerve fiber layer (RNFL) loss are considered as a biomarker for disease severity and progression [15–17]. Therefore, we aimed to perform a systematic review of the literature and meta-analysis to aggregate all of the existing data in determining the relationship between OCT measurements and Huntington’s disease as a neurodegenerative disorder.

## Materials and methods

The current study was conducted following the guidelines of the preferred reporting items for systematic reviews and meta-analyses (PRISMA) statement checklist, which provides an appropriate design for systematic reviews and meta-analyses [18]. Two authors (SF and MA) developed and registered the study protocol at the International Prospective Register of Systematic Reviews (PROSPERO) (Registration No. CRD42023433193).

### Data collection and extraction

Until June 7, 2023, a systematic literature search was carried out in three electronic databases (PubMed, Embase, Scopus), using the combination of terms “Huntington’s disease” and “optical coherence tomography” and their synonyms and related keywords (Table S1) to find studies reporting OCT measurements in patients with HD compared to healthy controls (HC). The reference sections of the studies were also explored to uncover related papers that were not discovered through database search. The main research was conducted by two authors (SF and MA). Both SF and MA screened all retrieved articles based on title, abstract, and full text. Two reviewers (MG and AM) independently extracted the following data from each of the included articles: first author’s name; publication year; location of study; design of the study; OCT model; method of eye selection (single eye, both eye, and mixed); inclusion and exclusion criteria of cases and non-cases; details of study populations, including sample size, mean and median age, percentage of males, mean intraocular pressure, mean axial length, and best corrected visual acuity measured by the logarithm of the minimum angle of resolution (LogMAR) in both groups of cases and controls; utilized diagnostic criteria for HD, HD scoring exams, disease duration in cases; outcome parameters of OCT measurements of various retinal layers and regions in the average values and, if available, the value of each sub-segment of the measured area in both cases and controls. Disagreements were resolved by discussion.

### Quality assessment

Two separate authors (MG and AM) evaluated the methodological quality of the included studies using the Newcastle–Ottawa scale (NOS), which is endorsed by the Cochrane Network [19]. The NOS rating scale is designed to evaluate the quality of case–control and cohort studies based on three criteria: selection, comparability, and exposure (in case–control studies) or outcome (in cohort studies). The total score for cohort studies is considered nine, while for case–control studies, it is eight. Disputes were solved through discussion.

### Statistical analysis

Stata version 16 software (StataCorp, College Station, TX) was used to perform meta-analysis on any SD-OCT metric, which was reported by at least three distinct studies. Weighted mean difference (WMD) and standardized mean difference (SMD), which was calculated by Hedges’ *g*, with a 95% confidence interval (CI) were utilized to pool the mean differences between compared groups. The MD is calculated by taking the difference between the means of the treatment group and the control group, while the SMD is the MD divided by the standard deviation (SD), derived from either or both of the groups [20]. Statistical heterogeneity was measured and the best analysis model was selected using Cochrane's *Q* and Higgins *I*^2^ tests. The fixed-effects model (*I*^2^ < 40%) or random-effects model was employed accordingly. Moreover, Egger’s test and the funnel plot were applied to identify potential publication bias, in which case the trim-and-fill method was conducted to adjust the effect size for any bias found [21, 22]. Finally, subgroup analysis and meta-regression were done to explore the effect of potential confounding variables. A *p* value lower than 0.05 was considered to be statistically significant.

## Results

### Study selection

**Fig. 1:**
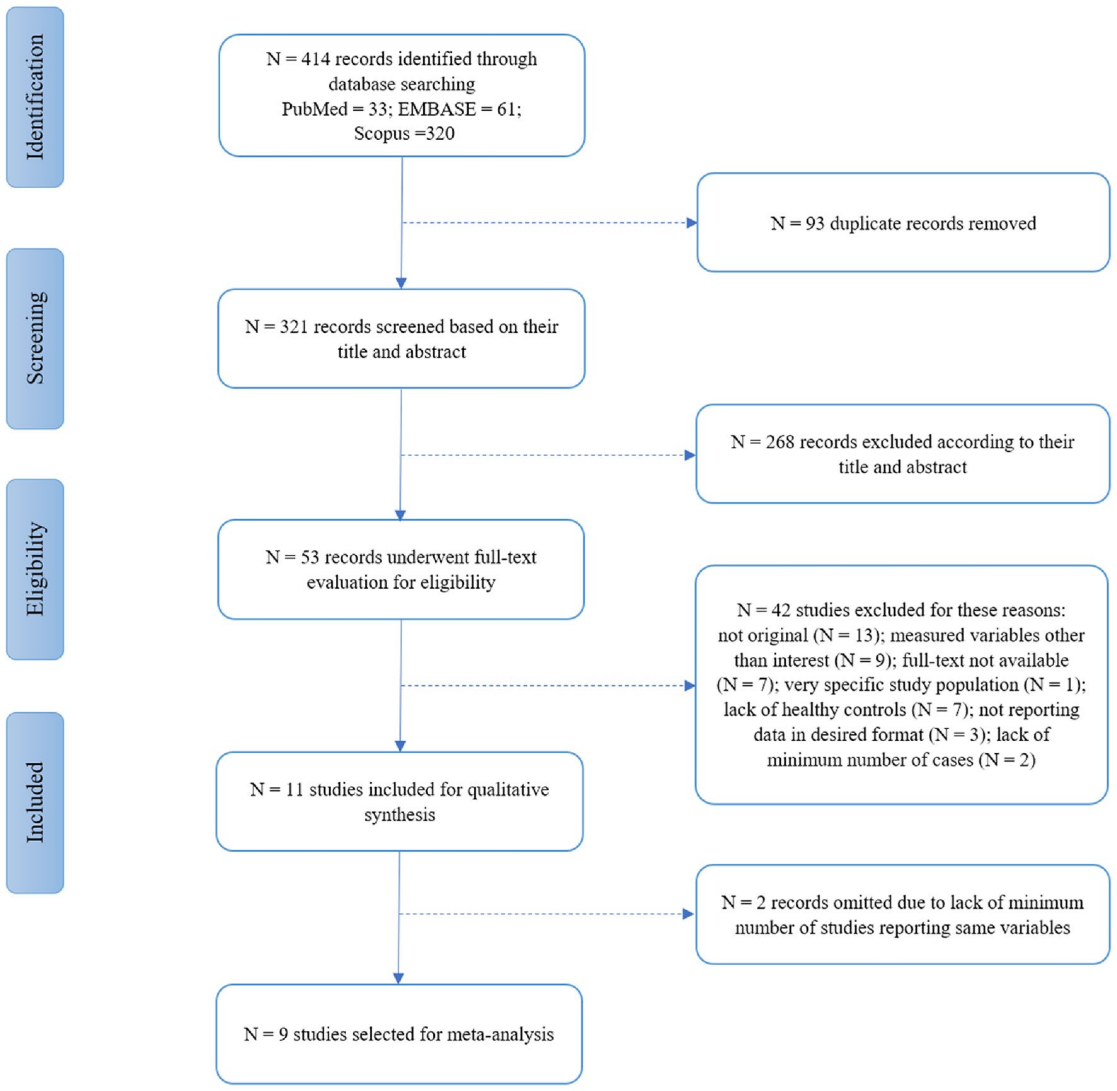
Flowchart of the study selection process

The complete stages of the study selection process are illustrated in Fig. 1. In the initial search of electronic databases, 414 records (33 in PubMed, 320 in Scopus, and 61 in Embase) were retrieved. Duplicate studies were automatically and manually identified and removed, and the remaining 321 studies were screened based on their title and abstract, excluding 268 irrelevant studies. In the final step, 53 full-text manuscripts were evaluated to identify eligible ones for inclusion. Forty-two articles were excluded in this phase due to the reasons brought in Fig. 1. Eleven studies were included in the systematic review [1, 3–5, 13, 23–28]. Two studies were not included in the meta-analysis as they did not report metrics in mean ± SD format reported in at least three of the included studies [3, 28]. A total population of 452 participants (241 cases, and 211 HC) was evaluated in nine studies, comprising 400 eyes with HD, 67 eyes of them reported specifically as pre-HD, and 349 healthy control (HC) eyes were included for meta-analysis. The studies by Murueta-Goyena et al. and Svetozarskiv et al. reported data of patients with HD in two separate groups (pre-HD and manifest HD), of which measurements of the manifest HD group were used for HD vs. HC analyses [23, 27]. Furthermore, Gatto et al. did not report the data for the whole HD group [4]; thus, the stage II HD subgroup with the highest number of cases was selected for HD vs. HC analyses.

### Characteristics of studies

The main characteristics of the included studies published from 2015 to 2023 were compiled in Table 1. All the studies had a case–control design, of which eight matched cases and controls for age and gender [1, 3–5, 13, 24, 25, 27]. Six studies declared patients with HD as their case group [1, 4, 5, 24–26]. Two studies only had a pre-HD group [3, 28], and three provided data for cases with pre-HD and manifest HD separately [13, 23, 27]. Regarding study location, eight studies were conducted in Europe [1, 3, 5, 23, 24, 26–28], and the other three studies were performed in Iran [25], Argentina [4], and New Zealand [13]. The mean age of study subjects ranged from 30.63 to 57.31 in the cases and from 35.75 to 55 in the controls. As the most used device, Heidelberg Spectralis (Heidelberg Engineering, Heidelberg, Germany) was applied in eight studies [1, 4, 5, 13, 24, 26–28]. Three studies used Optovue RTVue (Optovue Inc., Freemont, CA) [23, 25, 26], and one study used Revo NX 110 (Optopol, Zawiercie, Poland) [3]. All studies measured the Unified Huntington's Disease Rating Scale‐Total Motor Score (UHDRS‐TMS) as a disease severity scale. Information regarding OCT measurement including quality control and size of the performed scans in each study is shown in Table 1. Only the study by Murueta-Goyena et al. used OSCAR-IB criteria for quality control of the images [27].

**Table 1:** Characteristics of included studies

Study (first author/publication year)	Number of participants	Number of eyes	Eye selection method	Mean age	Male percentage	OCT model	Disease severity scales	Image quality control	Thickness measurement	Macula grid	Optic nerve head grid	Average pRNFL	Superior pRNFL	Inferior pRNFL	Temporal pRNFL	Nasal pRNFL	Central macular thickness	Subfoveal CT
Dusek/2023	41 HD	94	M	50.6	51.22	Heidelberg Spectralis	UHDRS-TMS, UHDRS-TFC	No specific guideline	Automatic	NR	3.4 × 3.4 mm	R	NR	NR	R	NR	NR	NR
	41 Controls	82		NR	NR													
Murueta-Goyena/2023	16 Pre-HD	32	M	46	25	Heidelberg Spectralis	UHDRS-TMS	OSCAR-IB Criteria	Automatic	6 × 6 mm	NR	R	NR	NR	R	NR	NR	NR
	20 Manifest HD^a^	38		53.4	45													
	16 Controls for Pre-HD	32		46	25													
	20 Controls for Manifest HD^a^	40		53.2	45													
Amini/2022	25 HD	46	M	49.76	56	Optovue RTVue	UHDRS-TMS	No specific guideline	Automatic	6 × 6 mm	NR	NR	R	R	R	R	R	NR
	25 Controls	50		44.32	40													
Mazur-Michalek/2022^b^	13 Pre-HD	26	B	43.1	NR	Revo NX 110	UHDRS	NR	Automatic	7 × 7 mm	3.4 × 3.4 mm	NR	NR	NR	NR	NR	NR	NR
	14 Controls	28		39.9	NR													
Schmid/2021^b^	24 Pre-HD	48	B	39.66	41.7	Heidelberg Spectralis	UHDRS-TMS, UHDRS cognitive score	No specific guideline	Automatic	6 × 6 mm	NR	NR	NR	NR	NR	NR	NR	NR
	38 Controls	76		35.75	36.8													
Di Maio/2020	16 HD	32	B	57.31	81.25	Heidelberg Spectralis and Optovue RTVue	UHDRS-TMS, UHDRS-TFC	No specific guideline	Automatic	7 × 7 mm	3.45 × 3.45 mm	R	R	R	NR	NR	NR	R
	13 Controls	26		55	61.54													
Svetozarskiv/2020	29 Pre-HD	29	S	30.63	NR	Optovue RTVue	UHDRS-TMS	NR	Manual	NR	3.46 × 3.46 mm	R	R	R	R	R	NR	R
	31 Manifest HD^a^	31		42.6	NR													
	31 Controls	31		37.3	NR													
Gatto/2018	14 HD	27	M	48.08	35.71	Heidelberg Spectralis	UHDRS-TMS	NR	Automatic	NR	NR	R	R	R	R	R	NR	NR
	3 Stage I HD			NR	NR													
	6 Stage II HD^a^			NR	NR													
	5 Stage III HD			NR	NR													
	13 Controls	26		NR	NR													
Gulmez Sevim/2018	15 HD	30	B	48	60	Heidelberg Spectralis	UHDRS-TMS, UHDRS-TFC, UHDRS independence score, Disease burden score	NR	Automatic	6 × 6 mm	3.4 × 3.4 mm	R	NR	NR	R	R	R	NR
	15 Controls	30		48	60													
Andrade/2016	8 HD	15	M	49.13	37.5	Heidelberg Spectralis	UHDRS-TMS, UHDRS-TFC, UHDRS independence score	NR	Automatic	6 × 6 mm	NR	R	R	R	R	R	R	R
	8 Controls	16		49.75	37.5													
Kersten/2015	26 HD	26	S	52.04	50	Heidelberg Spectralis	UHDRS-TMS, Disease burden score	No specific guideline	Automatic	6 × 6 mm	3.4 × 3.4 mm	R	R	R	R	R	R	NR
	6 Pre-HD	6		46	33.3													
	20 Manifest HD	20		53.85	55													
	29 Controls	29		50.69	37.9													

*HD* Huntington’s disease, *Pre-HD* pre-manifest Huntington’s disease, *OCT* optical coherence tomography, *UHDRS-TMS* The Unified Huntington's Disease Rating cale Total Motor Score, *UHDRS-TFC* The Unified Huntington's Disease Rating Scale Total Functional Capacity, *CT* choroidal thickness, *S* measurements of either single eye from each subject were selected, *B* measurements of both eyes from each subject were selected, *M* measurements of either single eye or both eyes from each subject were selected, *R* reported in the study, *NR* not reported in the study

^a^Measurements of this group was used for HD vs. control meta-analyses

^b^This study was not included in meta-analysis

### PRNFL thickness

#### Average pRNFL thickness

**Fig. 2:**
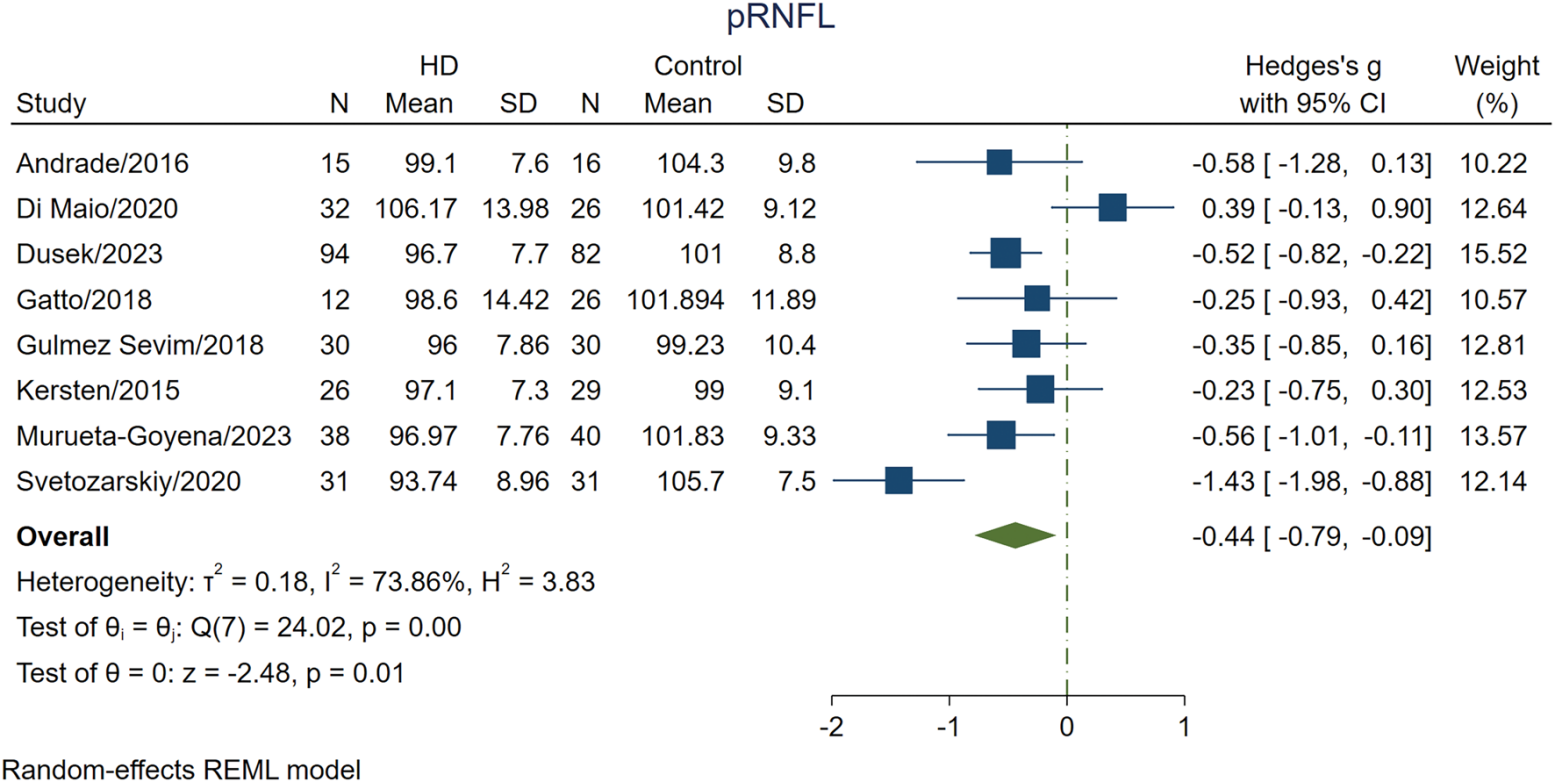
The difference in the average pRNFL thickness between patients with HD and healthy controls. The meta-analysis was conducted with a random-effects model. The size of the square for each article demonstrates the attributed weight, and the horizontal line indicates the 95% confidence interval (CI). The diamond shows the standardized mean difference, and its width represents the 95% CI. *HD* Huntington's disease, *N* number of subjects, *pRNFL* peripapillary retinal nerve fiber layer, *SD* standard deviation

Average pRNFL thickness was measured by eight studies [1, 4, 5, 13, 23, 24, 26, 27]. Results of the analysis indicated that patients with HD had significantly thinner average pRNFL than HC (SMD, − 0.44; 95% CI, − 0.79 to − 0.09; *p* = 0.0130; *I*^2^ = 73.86%; Table 2; Fig. 2). However, subjects with pre-HD had insignificant differences in average pRNFL thickness with the HC group (*p* = 0.44; Table 3). Mazur-Michalek et al. also found that the average pRNFL thickness was significantly reduced (*p* < 0.05) in both the left and right eyes of pre-HD in comparison with HC, but they did not provide quantitative data to be included in the meta-analysis [3].

**Table 2:** Differences in OCT measurements between patients with HD and controls

Variable	Overall effect				Heterogeneity		Egger’s test (*P* value)
	Standardized mean difference (95% CI)	*p* value	Weighted mean difference (95% CI)	*p* value	*I*^2^ test (%)	*Q* test (*p* value)	
Average pRNFL thickness	− 0.44 (− 0.79 to − 0.09)	**0.0130**	− 4.05 (− 7.17 to − 0.92)	**0.0111**	73.86	**0.0011**	0.99
Superior pRNFL thickness	− 0.05 (− 0.4 to 0.3)	0.78	− 0.84 (− 6.43 to − 4.75)	0.77	60.58	**0.0289**	0.71
Inferior pRNFL thickness	− 0.24 (− 0.60 to 0.12)	0.20	− 4.33 (− 10.55 to 1.90)	0.17	63.41	**0.0180**	0.29
Temporal pRNFL thickness	− 0.67 (− 1.07 to − 0.26)	**0.0012**	− 7.72 (− 12.42 to − 3.02)	**0.0013**	81.44	** < 0.0001**	0.42
Nasal pRNFL thickness	− 0.25 (− 0.55 to − 0.05)	0.10	− 3.68 (− 7.80 to 0.43)	0.08	46.05	0.09	0.92
Central macular thickness	− 0.21 (− 0.46 to 0.04)	0.10	− 4.17 (− 9.13 to 0.79)	0.10	0.00	0.47	0.85
Subfoveal CT	− 1.15 (− 1.49 to − 0.80)	** < 0.0001**	− 59.65 (− 76.37 to − 42.92)	** < 0.0001**	0.00	0.39	0.51

Boldface values indicate significance of the 95% confidence limit

*pRNFL* peripapillary retinal nerve fiber layer, *OCT* optical coherence tomography, *HD* Huntington’s disease, *CI* confidence interval, *CT* choroidal thickness

**Table 3:** Differences in OCT measurements between patients with pre-HD and controls

Variable	Overall effect				Heterogeneity		Egger’s test (*p* value)
	Standardized mean difference (95% CI)	*P* value	Weighted mean difference (95% CI)	*p* value	*I*^2^ test (%)	*Q* test (*p* value)	
Average pRNFL thickness	− 0.24 (− 0.86 to 0.37)	0.44	− 2.07 (− 7.29 to 3.17)	0.44	68.43	**0.0293**	0.87
Temporal pRNFL thickness	− 0.67 (− 2.02 to 0.67)	0.33	− 7.04 (− 20.39 to 6.32)	0.30	92.60	** < 0.0001**	0.69

Boldface values indicate significance of the 95% confidence limit

*pRNFL* peripapillary retinal nerve fiber layer, *OCT* optical coherence tomography, *pre-HD* pre-manifest Huntington’s disease, *CI* confidence interval

#### Superior and inferior pRNFL thickness

Six studies reported superior and inferior pRNFL thickness [4, 13, 23–26]. The differences for superior and inferior pRNFL thickness were not statistically significant between patients with HD and HC groups (all *p* > 0.05; Table 2). No analysis was conducted for pre-HD vs. HC since only two studies reported these metrics in pre-HD cases [13, 23]. Mazur-Michalek et al. [3] reported that the superior and inferior pRNFL thickness in both the left and right eyes of pre-HD were significantly less than HC (all *p* < 0.05). However, Svetozarskiy et al. [23] showed no significant difference in these two metrics between pre-HD and HC groups (all *p* > 0.05).

#### Temporal and nasal pRNFL thickness

**Fig. 3:**
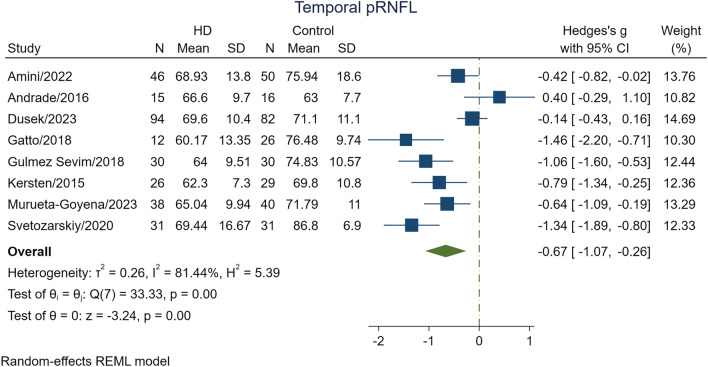
The difference in the temporal pRNFL thickness between patients with HD and healthy controls. The meta-analysis was conducted with a random-effects model. The size of the square for each article demonstrates the attributed weight, and the horizontal line indicates the 95% confidence interval (CI). The diamond shows the standardized mean difference, and its width represents the 95% CI. *HD* Huntington's disease, *N* number of subjects, *pRNFL* peripapillary retinal nerve fiber layer, *SD* standard deviation

Results of analysis on eight studies [1, 4, 5, 13, 23–25, 27] reporting temporal pRNFL thickness indicated that HD cases had significantly reduced temporal pRNFL thickness compared to the HC group (SMD − 0.67; 95% CI − 1.07 to − 0.26; *p* = 0.0012; *I*^2^ = 81.44%; Table 2; Fig. 3). However, a meta-analysis of three studies [13, 23, 27] reporting temporal pRNFL thickness in pre-HD cases showed a statistically insignificant difference in this variable compared to HC (*p* = 0.33; Table 3).

We identified six studies reporting nasal pRNFL in HD cases [4, 5, 13, 23–25]. The analysis demonstrated that no significant difference between HD and healthy eyes (*p* = 0.10; Table 2). This metric between pre-HD and HC was not analyzed since only two studies reported nasal pRNFLin pre-HD cases [13, 23]. Mazur-Michalek et al. [3] and Svetozarskiv et al. [23] both reported insignificant differences between pre-HD and healthy eyes in nasal pRNFL (all *p* > 0.05).

### Central macular thickness

Four studies were reporting central macular thickness [5, 13, 24, 25], the analysis of which did not show any significant difference between HD cases and HC (*p* = 0.10; Table 2). Only one study reported central macular thickness for pre-HD [13]; thus, no pre-HD vs. HC analysis was conducted for this variable.

### Subfoveal choroidal thickness

**Fig. 4:**
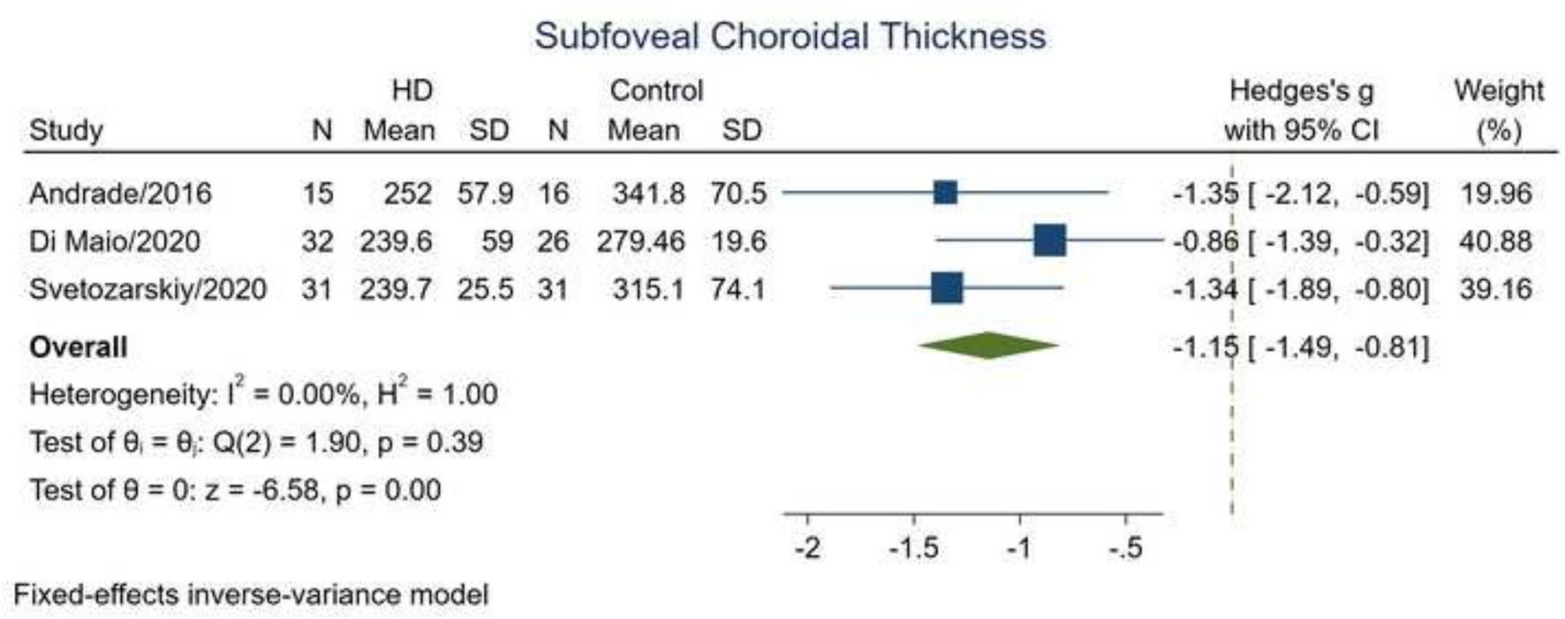
The difference in the subfoveal choroidal thickness between patients with HD and healthy controls. The meta-analysis was conducted with a random-effects model. The size of the square for each article demonstrates the attributed weight, and the horizontal line indicates the 95% confidence interval (CI). The diamond shows the standardized mean difference, and its width represents the 95% CI. *HD* Huntington's disease, *N* number of subjects, *SD* standard deviation

Three studies compared subfoveal choroidal thickness between patients with HD and HC [23, 24, 26]. Analysis of these studies denoted that subfoveal choroid had significantly reduced thickness in HD eyes in comparison to healthy eyes (SMD − 1.15; 95% CI − 1.49 to − 0.80; *p* < 0.0001; *I*^2^ = 0.00%; Table 2; Fig. 4). Only the study by Svetozarskiv et al. [23] reported subfoveal choroidal thickness in pre-HD cases separately and indicated that pre-HD participants had significantly thinner subfoveal choroid than HC (*p* = 0.006).

### Subgroup analysis

Wherever the heterogeneity was high and the number of studies allowed, we performed subgroup analyses according to variables that could have possible confounding effects on the observed differences. The subgroup analysis was done to compare average, superior, inferior, temporal, and nasal pRNFL between HD cases and the HC group.

#### Comparability of cases and controls

Results of subgroup analysis according to matching of cases and controls revealed a couple of inconsistent differences in subgroups compared to the overall findings (Table S2). The subset of studies that did not match (*p* = 0.43) cases and controls for age and sex had insignificant differences in average pRNFL between study groups, contrary to the results of the overall analysis and the subgroup with matching. Moreover, articles that matched study subjects for age and sex were the only subgroup in which HD cases showed significantly reduced inferior pRNFL thickness compared to HC (SMD − 0.41; 95% CI − 0.71 to − 0.12; *p* = 0.0065).

#### OCT model

We explored the possible effect of the OCT device used in the studies by the subgroup analysis (Table S3). Studies that used Optovue RTVue as their OCT model had incompatible results with the overall analyses for inferior, temporal, and nasal pRNFL. HD cases had significantly thinner inferior (SMD − 0.66; 95% CI − 0.98 to − 0.34; *p* < 0.0001) and nasal pRNFL (SMD − 0.48; 95% CI − 0.82 to − 0.15; *p* = 0.0050) than HC in the studies that applied Optovue RTVue. However, temporal pRNFL thickness was not significantly different between HD and healthy eyes in the Optovue RTVue subgroup (*p* = 0.06).

#### Study location

Subgroup analysis of average pRNFL thickness according to the study continent revealed insignificant differences in Oceania and South America subgroups (all *p* > 0.05; Table S4). Surprisingly, the subgroup of studies conducted in Europe, which composed the majority of studies, had borderline insignificant results in the analysis of temporal pRNFL thickness, unlike the overall significance (*p* = 0.06; Table S4). Nevertheless, the only study performed in Asia reported significantly reduced inferior pRNFL thickness, which was not in line with the overall result (SMD − 0.57; 95% CI − 0.97 to − 0.16; *p* = 0.0058; Table S4).

#### Eye selection method

The included studies had different eye selection methods, which could be a source of heterogeneity. Subgroup analysis based on the method of eye selection, revealed that, unlike the overall analysis, studies recruiting a single eye of patients for measurement and those with mixed methods showed insignificant differences in average pRNFL thickness between HD cases and HC (all *p* > 0.05; Table S5). Furthermore, the subgroup of studies with mixed methods of eye selection indicated no significant difference in temporal pRNFL thickness between HD and HC eyes, contrary to the overall results of the other subgroups (*p* = 0.10; Table S5).

### Meta-regression

Univariate meta-regression was evaluated to investigate the possible influence of suspected confounders on the findings of the meta-analysis. Thus, the effect of sample, male percentage, mean age, mean disease duration, mean gene repeats, and disease severity scores of cases on average, superior, inferior, temporal, and nasal pRNFL thickness analysis between HD and HC was examined (Table S6).

Effect sizes of analysis on average pRNFL thickness were revealed to have a significant direct correlation with male percentage (*β* 0.024; *p* = 0.003), mean age (*β* 0.101; *p* < 0.001), and mean BCVA of cases (*β* 22.429; *p* = 0.030) as well as a significant inverse correlation with mean disease duration (*β* − 0.100; *p* = 0.005). Differences detected in the analysis of superior pRNFL thickness were shown to have a significant positive correlation with the mean age of cases (*β* 0.078; *p* = 0.001) and significant negative correlation with mean IOP (*β* − 0.139; *p* = 0.015) and UHDRS-TMS of cases (*β* − 0.113; *p* = 0.041). Moreover, the only variable with a significant correlation with the analysis results on inferior pRNFL thickness was the mean age of cases (*β* 0.074; *p* = 0.003). Eventually, the mean UHDRS-TMS of cases was shown to have a significant inverse correlation with the effect sizes of analysis on nasal pRNFL thickness (*β* − 0.048; *p* = 0.037).

### Sensitivity analysis

Since there was significant heterogeneity in the conducted meta-analyses, we carried out sensitivity analyses by excluding the studies one at a time to identify studies that might be the root of the detected heterogeneity. Results of the analyses revealed that the exclusion of the study by Di Maio et al. [26] led to significantly thinner inferior pRNFL for HD patients than HC (SMD − 0.37; 95% CI − 0.71 to − 0.02; *p* = 0.036; Table S7). Likewise, the exclusion of the study by Gatto et al. [4] made the difference in nasal pRNFL thickness between HD and HC eyes statistically significant (SMD − 0.33; 95% CI − 0.62 to − 0.04; *p* = 0.025; Table S7). In the analysis of central macular thickness, HD cases showed significantly lower values compared to HC upon exclusion of the study by Kersten et al. [13] (SMD − 0.31; 95% CI − 0.60 to − 0.03; *p* = 0.031; Table S7). Pre-HD vs. HC sensitivity analyses demonstrated the exclusion of the study by Murueta-Goyena et al. [27] resulted in the significantly reduced average (SMD − 0.60; 95% CI − 1.04 to − 0.16; *p* = 0.008) and temporal thickness (SMD − 1.37; 95% CI − 2.21 to − 0.53; *p* = 0.001; Table S8) in pre-HD eyes compared to HC.

### Quality assessment

As all included studies were case–control, NOS was used to evaluate articles in the three independent domains of case selection, comparison of study groups, and exposure. All of the included studies received three scores in the exposure domain. Moreover, all studies had the same quality in the selection domain, receiving two scores. Regarding comparability, other than three studies [23, 26, 28], the rest of the studies matched cases and controls for at least age and sex and received a maximum score of two. Therefore, the total score received by the included studies was 7 for eight studies and 5 for three studies (Table S9).

## Discussion

A connection has been established between ocular structural changes and various neurodegenerative and systemic disorders, such as AD, PD, MS, Systemic Lupus Erythematosus (SLE), and Rheumatoid Arthritis (RA) [29–31]. Hence, numerous studies utilizing different diagnostic tools, such as visual-evoked potential (VEP) and OCT, have detected signs of retinal structural alterations or a decline in the functionality of visual pathways among individuals with HD [7, 8, 10, 11]. The inconsistent results reported in the HD literature regarding alterations in these ocular layers encouraged us to conduct this systematic review and meta-analysis to accumulate and unify the findings. Results of the current study indicate that patients with HD exhibited significant thinning of the average and temporal pRNFL compared to HC. In contrast, subjects with pre-HD did not show any significant differences. Furthermore, there were no significant differences observed in superior and inferior pRNFL thickness between patients with HD and HC. Moreover, the subfoveal choroid was found thinner in HD patients compared to HC. However, the central macular thickness was not significantly different between HD and HC eyes.

The HD mutation found in the exon-1 of the huntingtin HTT gene is expressed in all types of cells and tissues comprising the eyes [32–34]. Researchers have used murine models with HD to investigate retinopathy associated with the condition, discovering notable alterations in the structure of the retina, such as increased retinal stress and dysfunction in addition to progressive neuronal remodeling and disorganization of the photoreceptor layer [35, 36]. Supporting this notion, the current meta-analysis showed that patients with HD had significant thinning in average and temporal pRNFL. The effect size of the difference for temporal pRNFL was the greatest. It was suggested that temporal pRNFL is the most susceptible quadrant of pRNFL to neurodegeneration [4]. Involvement of the optic nerve and loss of the temporal pRNFL, which plays a vital role in the color and central vision, is one of the prominent properties of neurodegenerative disorders with mitochondrial dysfunction, namely Friedreich’s ataxia and hereditary spastic paraplegia (SPG7) [13, 37].

Interestingly, the presence of mitochondrial dysfunction in HD through biochemical, imaging, and molecular investigations has been extensively reported [38, 39]. Moreover, studies have demonstrated that the mutant HTT hinders the transportation of mitochondria [13, 40]. This may explain the significant thinning of temporal pRNFL in eyes with HD. However, it should be noted that a postmortem case report by Petrasch-Parwez et al. demonstrated that the retina of HD case did not have any obvious macroscopical or histological alterations indicating degeneration and no inclusions or aggregates of HTT and ubiquitin were detected [41].

Furthermore, the central macular thickness was not significantly different in patients with HD compared to healthy individuals according to the findings of this study. Several inner cellular layers of the retina, such as the ganglion cell complex (GCC) and inner plexiform layer (IPL), were not the subject of meta-analysis due to an insufficient number of studies. However, several studies reported significant alterations in these layers. For instance, Svetozarskiy et al. [23] reported significantly thinned GCC in HD patients. GCC consists of ganglion cells, axons of which compose the optic nerve in RNFL. The aforementioned mitochondrial dysfunction could prompt substantial loss in this layer's high energy-consuming cells, leading to the observed diminished thickness [39, 42]. It should be noted that sensitivity analysis indicated that the exclusion of the study by Kersten et al. [13], which had pre-manifest patients in its study population, made the difference in central macular thickness statistically significant.

The individual studies by Mazur-Michalek et al. and Svetzoraskiy et al. reported that individuals with pre-HD exhibited a significant decrease in the average thickness of pRNFL and the thickness of temporal pRNFL [3, 23]. However, the outputs of the meta-analysis did not indicate any significant differences for these two variables. After the exclusion of the study by Murueta-Goyena et al. from analysis, eyes with HD showed significantly reduced average and temporal pRNFL thickness compared to HC [27]. The study by Murueta-Goyena et al. has shown no significant difference in OCT measurement between HD and HC groups except for external limiting membrane-Bruch’s membrane complex (ELM-BM). Therefore, as we were only able to perform meta-analysis on limited variables with a low number of studies, OCT might be considered not sensitive enough to be applied in the pre-HD population at least until further longitudinal investigations considering variables such as the duration between OCT measurement and disease onset or disease burden score.

Nonetheless, subfoveal choroidal thickness was also shown to be downsized in HD patients in comparison to HC. It has been well established that both the functional requirements and structural remodeling determine the thickness of the choroid as a highly dynamic vascular structure [43, 44]. OCT angiography (OCTA) studies reported and confirmed choroidal thinning in similar neurodegenerative diseases, such as PD and MS [15, 16]. The impairment of the blood–brain barrier mentioned in the previous studies could support the idea that cerebrovascular dysfunction plays a role in the development of HD [45, 46]. Moreover, the thinning of the macular choroid in HD can be observed due to disruptions in intracranial blood flow, which are probably associated with the abnormal buildup of huntingtin in the brain's blood vessels [46]. Moreover, this can subsequently cut down the nourishment served for retinal layers by choroid and lead to observed reduced thickness in these structures. Oppositely, cellular loss in the retina reducing the energy demand might dwindle the supply provided by the choroid kindling the decreased thickness in this vascular layer [47]. Andrade et al. observed that the thinning of macular choroid had no relation with motor score and disease severity suggesting that choroidal alterations occur prior to retinal changes [24]. Therefore, macular choroidal may be more suitable for diagnosis of presymptomatic patients whereas monitoring the progression of the disease could be more effectively done using the macular retina. When considering these results as a whole, addressing the neurovasculopathy in HD contributing to brain atrophy could be a potential therapeutic target [46].

Findings of the meta-regression conducted in the current review revealed that disease duration and UHDRS-TMS significantly correlated with the effect sizes of analysis on pRNFL thickness between HD and HC eyes. This is in line with the findings of several studies that reported a significant correlation between macular thickness and temporal pRNFL thickness with disease duration and UHDRS [27]. In a recent study, Amini et al. discovered a correlation between the duration of the disease and the overall thickness of the macula [25]. Similarly, Gulmez Sevim et al. [5] observed a significant correlation between the thickness of the macular ganglion cell-inner plexiform layer (GCIPL), disease duration, disease burden, CAG repeats, UHDRS, and patient independence in individuals with manifest HD. On the other hand, the studies by Murueta-Goyena et al. and Dusek et al. failed to find a significant correlation between retinal metrics and disease duration or UHDRS [1, 27]. Moreover, our meta-regression showed a significant correlation of HD cases' age with the effect sizes of difference in average, superior, and inferior pRNFL thickness but not temporal pRNFL. However, Dusek et al. [1] reported a significant effect of age in the intergroup analysis, suggesting their findings supported that temporal RNFL thinning is driven by age progression rather than the influence of disease and this could explain the discrepancy in the results of studies with different populations.

Although the current study is the first systematic review and meta-analysis of the OCT in patients with HD, it is important to acknowledge the limitations. The low number of eligible studies with relatively small sample sizes was the prime limitation. HD is an uncommon condition, and it is difficult to reach a broader population of patients who are interested in taking part in the research. Moreover, exclusively adult patients were study subjects with limited maximum CAG expansion length; thus, these results are not transferable to juvenile Huntington’s disease or late disease stages. Additionally, subgroup analysis presented that methodological varieties such as OCT device and eye selection method caused heterogeneity in existing studies, warranting further investigation by longitudinal studies with standard methodologies and more extended follow-up periods to establish the robustness and generalizability of OCT findings and assess the predictive value of OCT measurements concerning disease progression and cognitive decline. Furthermore, although most of the included studies checked the quality of images and excluded the results with motion artifacts which is common in OCT imaging [48], involuntary movements in the patients with HD might affect the quality of images and limit the clinical use of OCT in these individuals.

## Conclusion

Results of the current systematic review and meta-analysis confirmed the reports of previous studies on the significant thinning of average and temporal pRNFL and subfoveal choroid in patients with HD compared to HC. Despite the critical limitations, such as the lack of longitudinal studies with large populations, the findings from this study provided primary evidence that OCT metrics have the potential to serve as a non-invasive biomarker for assessing retinal changes in HD. Further research is needed to validate these findings, identify the underlying mechanisms, and establish OCT as a routine diagnostic tool in HD clinics. If successful, OCT could contribute to early detection, disease monitoring, and the developing novel therapeutic interventions for HD patients.

## Supplementary Information

Below is the link to the electronic supplementary material.

Supplementary file1 (DOCX 47 KB)
