# Supplementary material for: Optical coherence tomography measurements in Huntington’s disease: a systematic review and meta-analysis
Source: J Neurol. 2024 Aug 26;271(10):6471–84. doi: 10.1007/s00415-024-12634-4 (PMC11447008; doi:10.1007/s00415-024-12634-4)
Supplement: Supplementary file 1 — Supplementary file1 (DOCX 47 KB) [file 415_2024_12634_MOESM1_ESM.docx]

| **Table S1.** Search strategies for each database | |
| --- | --- |
| **Database** | **Search string** |
| PubMed | ((“Huntington Chorea” OR “Chorea, Huntington” OR “Huntington's Disease” OR “Chronic Progressive Hereditary Chorea (Huntington)” OR “Huntington Chronic Progressive Hereditary Chorea” OR “Progressive Chorea, Chronic Hereditary (Huntington)” OR “Progressive Chorea, Hereditary, Chronic (Huntington)” OR “Huntington's Chorea” OR “Chorea, Huntington's” OR “Chorea, Chronic Progressive Hereditary (Huntington)” OR “Huntington Disease, Late Onset” OR “Late-Onset Huntington Disease” OR “Huntington Disease, Late-Onset” OR “Late Onset Huntington Disease” OR “Juvenile Huntington Disease” OR “Juvenile-Onset Huntington Disease” OR “Juvenile Onset Huntington Disease” OR “Huntington Disease, Juvenile-Onset” OR “Huntington Disease, Juvenile Onset” OR “Huntington Disease, Juvenile” OR “Akinetic-Rigid Variant of Huntington Disease” OR “Akinetic Rigid Variant of Huntington Disease” OR “Huntington Disease, Akinetic-Rigid Variant” OR “Huntington Disease, Akinetic Rigid Variant”)) AND ("Tomography, Optical Coherence" OR "Coherence Tomography, Optical" OR "OCT Tomography" OR "Tomography, OCT" OR "Optical Coherence Tomography")) |
| EMBASE | ('huntington disease'/exp OR 'huntington disease' OR 'chronic progressive hereditary chorea' OR 'huntington chronic progressive hereditary chorea' OR 'progressive chorea, chronic hereditary' OR 'progressive chorea, hereditary, chronic' OR 'huntington chorea'/exp OR 'huntington chorea' OR 'chorea, huntington'/exp OR 'chorea, huntington' OR 'chorea, chronic progressive hereditary' OR 'huntington disease, late onset' OR 'late-onset huntington disease' OR 'huntington disease, late-onset' OR 'late onset huntington disease' OR 'juvenile huntington disease'/exp OR 'juvenile huntington disease' OR 'juvenile-onset huntington disease' OR 'juvenile onset huntington disease' OR 'huntington disease, juvenile-onset' OR 'huntington disease, juvenile onset' OR 'huntington disease, juvenile' OR 'akinetic-rigid variant of huntington disease' OR 'akinetic rigid variant of huntington disease' OR 'huntington disease, akinetic-rigid variant' OR 'huntington disease, akinetic rigid variant') AND ('tomography, optical coherence' OR 'coherence tomography, optical' OR 'oct tomography' OR 'tomography, oct' OR 'optical coherence tomography') |
| Scopus | ALL ((“Huntington Chorea” OR “Chorea, Huntington” OR “Huntington's Disease” OR “Chronic Progressive Hereditary Chorea (Huntington)” OR “Huntington Chronic Progressive Hereditary Chorea” OR “Progressive Chorea, Chronic Hereditary (Huntington)” OR “Progressive Chorea, Hereditary, Chronic (Huntington)” OR “Huntington's Chorea” OR “Chorea, Huntington's” OR “Chorea, Chronic Progressive Hereditary (Huntington)” OR “Huntington Disease, Late Onset” OR “Late-Onset Huntington Disease” OR “Huntington Disease, Late-Onset” OR “Late Onset Huntington Disease” OR “Juvenile Huntington Disease” OR “Juvenile-Onset Huntington Disease” OR “Juvenile Onset Huntington Disease” OR “Huntington Disease, Juvenile-Onset” OR “Huntington Disease, Juvenile Onset” OR “Huntington Disease, Juvenile” OR “Akinetic-Rigid Variant of Huntington Disease” OR “Akinetic Rigid Variant of Huntington Disease” OR “Huntington Disease, Akinetic-Rigid Variant” OR “Huntington Disease, Akinetic Rigid Variant”)) AND ("Tomography, Optical Coherence" OR "Coherence Tomography, Optical" OR "OCT Tomography" OR "Tomography, OCT" OR "Optical Coherence Tomography")) |

| **Table S2. Results of subgroup analysis based on matching cases and controls** | | | | | | |
| --- | --- | --- | --- | --- | --- | --- |
| **Variable** (Comparison groups) | **All Eligible Studies** | | **Studies that matched for age and sex** | | **Studies that did not match** | |
|  | **Standardized Mean Difference (95% CI)** | ***p* value** | **Standardized Mean Difference (95% CI)** | ***p* value** | **Standardized Mean Difference (95% CI)** | ***p* value** |
|  |  |  |  |  |  |  |
| **Average pRNFL thickness** (HD vs Controls) | -0.44 (-0.79 to -0.09) | **0.0130** | -0.46 (-0.66 to -0.27) | **<0.0001** | -0.43 (-1.49 to 0.63) | 0.43 |
|  |  |  |  |  |  |  |
| **Superior pRNFL thickness** (HD vs Controls) | -0.05 (-0.40 to 0.30) | 0.78 | 0.07 (-0.21 to 0.36) | 0.64 | -0.20 (-0.88 to 0.48) | 0.58 |
|  |  |  |  |  |  |  |
| **Inferior pRNFL thickness** (HD vs Controls) | -0.24 (-0.60 to 0.12) | 0.20 | -0.41 (-0.71 to -0.12) | **0.0065** | -0.08 (-0.82 to 0.65) | 0.84 |
|  |  |  |  |  |  |  |
| **Temporal pRNFL thickness** (HD vs Controls) | -0.67 (-1.07 to -0.26) | **0.0012** | -0.45 (-0.82 to -0.09) | **0.0156** | -1.38 (-1.82 to -0.94) | **<0.0001** |
|  |  |  |  |  |  |  |
| **Nasal pRNFL thickness** (HD vs Controls) | -0.25 (-0.55 to 0.05) | 0.10 | -0.23 (-0.49 to 0.03) | 0.08 | -0.22 (-1.20 to 0.75) | 0.67 |
|  |  |  |  |  |  |  |
| pRNFL, peripapillary retinal nerve fiber layer; HD, Huntington's disease; CI, confidence interval.  Boldface values indicate significance of the 95% confidence limit. | | | | | | |

| **Table S3. Results of subgroup analysis based on the OCT device** | | | | | | |
| --- | --- | --- | --- | --- | --- | --- |
| **Variable** (Comparison groups) | **All Eligible Studies** | | **Studies with** **Heidelberg Spectralis** | | **Studies with Optovue RTVue** | |
|  | **Standardized Mean Difference (95% CI)** | ***p* value** | **Standardized Mean Difference (95% CI)** | ***p* value** | **Standardized Mean Difference (95% CI)** | ***p* value** |
|  |  |  |  |  |  |  |
| **Average pRNFL thickness** (HD vs Controls) | -0.44 (-0.79 to -0.09) | **0.0130** | -0.31 (-0.57 to -0.06) | **0.0171** | -1.43 (-1.98 to -0.88) | **<0.0001** |
|  |  |  |  |  |  |  |
| **Superior pRNFL thickness** (HD vs Controls) | -0.05 (-0.40 to 0.30) | 0.78 | 0.13 (-0.26 to 0.52) | 0.52 | -0.32 (-0.90 to 0.26) | 0.28 |
|  |  |  |  |  |  |  |
| **Inferior pRNFL thickness** (HD vs Controls) | -0.24 (-0.60 to 0.12) | 0.20 | 0.03 (-0.29 to 0.35**)** | 0.86 | -0.66 (-0.98 to -0.34) | **<0.0001** |
|  |  |  |  |  |  |  |
| **Temporal pRNFL thickness** (HD vs Controls) | -0.67 (-1.07 to -0.26) | **0.0012** | -0.60 (-1.09 to -0.11) | **0.0163** | -0.86 (-1.77 to 0.04) | 0.06 |
|  |  |  |  |  |  |  |
| **Nasal pRNFL thickness** (HD vs Controls) | -0.25 (-0.55 to 0.05) | 0.10 | -0.08 (-0.43 to 0.26) | 0.66 | -0.48 (-0.82 to -0.15) | **0.0050** |
|  |  |  |  |  |  |  |
| pRNFL, peripapillary retinal nerve fiber layer, HD; Huntington's disease; OCT, optical coherence tomography; CI, confidence interval.  Boldface values indicate significance of the 95% confidence limit. | | | | | | |

| **Table S4. Results of subgroup analysis based on the study continent** | | | | | | | | | | |
| --- | --- | --- | --- | --- | --- | --- | --- | --- | --- | --- |
| **Variable** (Comparison groups) | **All Eligible Studies** | | **Studies conducted in Europe** | | **Studies conducted in Asia** | | **Studies conducted in Oceania** | | **Studies conducted in South America** | |
|  | **Standardized Mean Difference (95% CI)** | ***p* value** | **Standardized Mean Difference (95% CI)** | ***p* value** | **Standardized Mean Difference (95% CI)** | ***p* value** | **Standardized Mean Difference (95% CI)** | ***p* value** | **Standardized Mean Difference (95% CI)** | ***p* value** |
|  |  |  |  |  |  |  |  |  |  |  |
| **Average pRNFL thickness** (HD vs Controls) | -0.44 (-0.79 to -0.09) | **0.0130** | -0.50 (-0.96 to -0.05) | **0.0310** |  |  | -0.23 (-0.75 to 0.30) | 0.40 | -0.25 (-0.93 to 0.42) | 0.48 |
|  |  |  |  |  |  |  |  |  |  |  |
| **Superior pRNFL thickness** (HD vs Controls) | -0.05 (-0.40 to 0.30) | 0.78 | -0.09 (-0.74 to 0.56) | 0.80 | -0.05 (-0.44 to 0.35) | 0.82 | 0.37 (-0.16 to 0.89) | 0.17 | -0.43 (-1.11 to 0.24) | 0.21 |
|  |  |  |  |  |  |  |  |  |  |  |
| **Inferior pRNFL thickness** (HD vs Controls) | -0.24 (-0.60 to 0.12) | 0.20 | -0.26 (-0.91 to 0.39) | 0.44 | -0.57 (-0.97 to -0.16) | **0.0058** | -0.25 (-0.77 to 0.28) | 0.36 | 0.31 (-0.36 to 0.98) | 0.37 |
|  |  |  |  |  |  |  |  |  |  |  |
| **Temporal pRNFL thickness** (HD vs Controls) | -0.67 (-1.07 to -0.26) | **0.0012** | -0.56 (-1.15 to 0.02) | 0.06 | -0.42 (-0.82 to -0.02) | **0.0393** | -0.79 (-1.34 to -0.25) | **0.0045** | -1.46 (-2.20 to -0.71) | **0.0001** |
|  |  |  |  |  |  |  |  |  |  |  |
| **Nasal pRNFL thickness** (HD vs Controls) | -0.25 (-0.55 to 0.05) | 0.10 | -0.45 (-0.95 to 0.05) | 0.08 | -0.35 (-0.75 to 0.05) | 0.09 | -0.02 (-0.54 to 0.50) | 0.95 | 0.30 (-0.37 to 0.97) | 0.39 |
|  |  |  |  |  |  |  |  |  |  |  |
| pRNFL, peripapillary retinal nerve fiber layer; HD, Huntington's disease; CI, confidence interval.  Boldface values indicate significance of the 95% confidence limit. | | | | | | | | | | |

| **Table S5. Results of subgroup analysis based on the method of eye selection** | | | | | | | | |
| --- | --- | --- | --- | --- | --- | --- | --- | --- |
| **Variable** (Comparison groups) | **All Eligible Studies** | | **Studies that measured both eyes** | | **Studies that measured single eye** | | **Studies with mixed method** | |
|  | **Standardized Mean Difference (95% CI)** | ***p* value** | **Standardized Mean Difference (95% CI)** | ***p* value** | **Standardized Mean Difference (95% CI)** | ***p* value** | **Standardized Mean Difference (95% CI)** | ***p* value** |
|  |  |  |  |  |  |  |  |  |
| **Average pRNFL thickness** (HD vs Controls) | -0.44 (-0.79 to -0.09) | **0.0130** | 0.02 (-0.70 to 0.74) | 0.96 | -0.82 (-2.00 to 0.36) | 0.17 | -0.51 (-0.73 to -0.28) | **<0.0001** |
|  |  |  |  |  |  |  |  |  |
| **Superior pRNFL thickness** (HD vs Controls) | -0.05 (-0.40 to 0.30) | 0.78 | 0.45 (-0.07 to 0.97) | 0.09 | -0.14 (-1.13 to 0.85) | 0.79 | -0.13 (-0.44 to 0.18) | 0.42 |
|  |  |  |  |  |  |  |  |  |
| **Inferior pRNFL thickness** (HD vs Controls) | -0.24 (-0.60 to 0.12) | 0.20 | 0.29 (-0.22 to 0.80) | 0.27 | -0.53 (-1.07 to 0.02) | 0.06 | -0.23 (-0.74 to 0.29) | 0.39 |
|  |  |  |  |  |  |  |  |  |
| **Temporal pRNFL thickness** (HD vs Controls) | -0.67 (-1.07 to -0.26) | **0.0012** | -1.06 (-1.60 to -0.53) | **0.0001** | -1.07 (-1.61 to -0.53) | **0.0001** | -0.43 (-0.95 to 0.09) | 0.10 |
|  |  |  |  |  |  |  |  |  |
| **Nasal pRNFL thickness** (HD vs Controls) | -0.25 (-0.55 to 0.05) | 0.10 | 0.01 (-0.49 to 0.51) | 0.97 | -0.36 (-1.02 to 0.30) | 0.29 | -0.27 (-0.80 to 0.26) | 0.32 |
|  |  |  |  |  |  |  |  |  |
| pRNFL, peripapillary retinal nerve fiber layer; HD, Huntington's disease; CI, confidence interval.  Boldface values indicate significance of the 95% confidence limit. | | | | | | | | |

| **Table S6. Results of univariate meta-regression on OCT measurements** | | | | | | | | | | | | | | | | | | |
| --- | --- | --- | --- | --- | --- | --- | --- | --- | --- | --- | --- | --- | --- | --- | --- | --- | --- | --- |
| **Variable** (Comparison groups) | **Sample Size** | | **Male Percentage of Cases** | | **Mean Age of Cases** | | **Mean BCVA of Cases** | | **Mean IOP of Cases** | | **Mean Disease Duration** | | **Mean Gene Repeat** | | **Mean UHDRS-TMS** | | **Mean UHDRS-TFC** | |
|  | **β coefficient** | **p value** | **β coefficient** | **p value** | **β coefficient** | **p value** | **β coefficient** | **p value** | **β coefficient** | **p value** | **β coefficient** | **p value** | **β coefficient** | **p value** | **β coefficient** | **p value** | **β coefficient** | **p value** |
|  |  |  |  |  |  |  |  |  |  |  |  |  |  |  |  |  |  |  |
| **Average pRNFL thickness** (HD vs Controls) | -0.0011463 | 0.789 | 0.0238369 | **0.003** | 0.1011984 | **<0.001** | 22.42888 | **0.030** |  |  | -0.0999292 | **0.005** | -0.5152484 | 0.193 | -0.0583981 | 0.231 | 0.1115319 | 0.556 |
|  |  |  |  |  |  |  |  |  |  |  |  |  |  |  |  |  |  |  |
| **Superior pRNFL thickness** (HD vs Controls) | 0.0012244 | 0.897 | 0.0098821 | 0.305 | 0.078212 | **0.001** | 9.243067 | 0.148 | -0.1387335 | **0.015** |  |  | -0.1164873 | 0.585 | -0.1132811 | **0.041** |  |  |
|  |  |  |  |  |  |  |  |  |  |  |  |  |  |  |  |  |  |  |
| **Inferior pRNFL thickness** (HD vs Controls) | -0.0096324 | 0.257 | 0.015265 | 0.179 | 0.073602 | **0.003** | 4.568713 | 0.543 | -0.0677082 | 0.220 |  |  | -0.0001776 | 0.999 | -0.0880968 | 0.306 |  |  |
|  |  |  |  |  |  |  |  |  |  |  |  |  |  |  |  |  |  |  |
| **Temporal pRNFL thickness** (HD vs Controls) | 0.0043101 | 0.347 | -0.0428347 | 0.081 | 0.0743611 | 0.226 | -0.8872093 | 0.952 | -0.1327199 | 0.061 |  |  | -0.1372433 | 0.568 | 0.0087704 | 0.882 |  |  |
|  |  |  |  |  |  |  |  |  |  |  |  |  |  |  |  |  |  |  |
| **Nasal pRNFL thickness** (HD vs Controls) | -0.0018995 | 0.817 | 0.0247478 | 0.201 | 0.0575607 | 0.155 | 7.004182 | 0.120 | -0.0875855 | 0.109 | -0.0574521 | 0.128 | -0.0590103 | 0.721 | -0.0478891 | **0.037** |  |  |
|  |  |  |  |  |  |  |  |  |  |  |  |  |  |  |  |  |  |  |
| pRNFL, peripapillary retinal nerve fiber layer; HD, Huntington's disease; OCT, optical coherence tomography; BCVA, best corrected visual acuity; IOP, intraocular pressure; UHDRS-TMS, The Unified Huntington's Disease Rating Scale Total Motor Score; UHDRS-TFC, The Unified Huntington's Disease Rating Scale Total Functional Capacity.  Boldface values indicate significance of the 95% confidence limit. | | | | | | | | | | | | | | | | | | |

| **Table S7. Sensitivity analysis of OCT measurements difference between patients with HD and controls** | | | |
| --- | --- | --- | --- |
| **Variable** | **Omitted Study**  **(First Author/Publication Year)** | **New Standardized Mean Difference (95% CI)** | ***P* Value** |
|  |  |  |  |
| Average pRNFL Thickness | Dusek/2023 | -0.43 (-0.84 to -0.01) | **0.043** |
|  | Murueta-Goyena/2023 | -0.42 (-0.82 to -0.02) | **0.041** |
|  | Di Maio/2020 | -0.56 (-0.84 to -0.27) | **<0.001** |
|  | Svetozarskiy/2020 | -0.31 (-0.57 to -0.06) | **0.015** |
|  | Gatto/2018 | -0.46 (-0.85 to -0.07) | **0.020** |
|  | Gulmez Sevim/2018 | -0.45 (-0.85 to -0.05) | **0.027** |
|  | Andrade/2016 | -0.42 (-0.81 to -0.03) | **0.034** |
|  | Kersten/2015 | -0.47 (-0.87 to -0.07) | **0.020** |
|  |  |  |  |
| Superior pRNFL Thickness | Amini/2022 | -0.05 (-0.50 to 0.39) | 0.81 |
|  | Di Maio/2020 | -0.15 (-0.51 to 0.20) | 0.40 |
|  | Svetozarskiy/2020 | 0.09 (-0.20 to 0.37) | 0.55 |
|  | Gatto/2018 | 0.01 (-0.38 to 0.40) | 0.96 |
|  | Andrade/2016 | -0.05 (-0.46 to 0.36) | 0.82 |
|  | Kersten/2015 | -0.14 (-0.52 to 0.24) | 0.48 |
|  |  |  |  |
| Inferior pRNFL  Thickness | Amini/2022 | -0.16 (-0.58 to 0.27) | 0.47 |
|  | Di Maio/2020 | -0.37 (-0.71 to -0.02) | **0.036** |
|  | Svetozarskiy/2020 | -0.13 (-0.49 to 0.23) | 0.49 |
|  | Gatto/2018 | -0.33 (-0.70 to 0.04) | 0.08 |
|  | Andrade/2016 | -0.23 (-0.66 to 0.20) | 0.30 |
|  | Kersten/2015 | -0.23 (-0.68 to 0.22) | 0.31 |
|  |  |  |  |
| Temporal pRNFL  Thickness | Dusek/2023 | -0.76 (-1.19 to -0.33) | **0.001** |
|  | Murueta-Goyena/2023 | -0.67 (-1.5 to -0.20) | **0.005** |
|  | Amini/2022 | -0.71 (-1.17 to -0.24) | **0.003** |
|  | Svetozarskiy/2020 | -0.57 (-0.97 to -0.17) | **0.006** |
|  | Gatto/2018 | -0.58 (-0.98 to -0.17) | **0.005** |
|  | Gulmez Sevim/2018 | -0.61 (-1.06 to -0.16) | **0.007** |
|  | Andrade/2016 | -0.78 (-1.14 to -0.42) | **<0.001** |
|  | Kersten/2015 | -0.65 (-1.12 to -0.18) | **0.006** |
|  |  |  |  |
| Nasal pRNFL  Thickness | Amini/2022 | -0.23 (-0.62 to 0.16) | 0.25 |
|  | Svetozarskiy/2020 | -0.16 (-0.43 to 0.11) | 0.25 |
|  | Gatto/2018 | -0.33 (-0.62 to -0.04) | **0.025** |
|  | Gulmez Sevim/2018 | -0.31 (-0.66 to 0.04) | 0.08 |
|  | Andrade/2016 | -0.19 (-0.5 to 0.12) | 0.24 |
|  | Kersten/2015 | -0.30 (-0.65 to 0.05) | 0.10 |
|  |  |  |  |
| Central macular Thickness | Amini/2022 | -0.12 (-0.44 to 0.20) | 0.47 |
|  | Gulmez Sevim/2018 | -0.21 (-0.50 to 0.08) | 0.15 |
|  | Andrade/2016 | -0.18 (-0.45 to 0.09) | 0.19 |
|  | Kersten/2015 | -0.31 (-0.60 to -0.03) | **0.031** |
|  |  |  |  |
| Subfoveal CT | Di Maio/2020 | -1.35 (-1.79 to -0.90) | **<0.001** |
|  | Svetozarskiy/2020 | -1.02 (-1.46 to -0.58) | **<0.001** |
|  | Andrade/2016 | -1.10 (-1.48 to -0.71) | **<0.001** |
|  |  |  |  |
| pRNFL, peripapillary retinal nerve fiber layer; OCT, optical coherence tomography; HD, Huntington’s disease; CI, confidence interval; CT, choroidal thickness.  Boldface values indicate significance of the 95% confidence limit. | | | |

| **Table S8. Sensitivity analysis of OCT measurements difference between patients with pre-HD and controls** | | | |
| --- | --- | --- | --- |
| **Variable** | **Omitted Study**  **(First Author/Publication Year)** | **New Standardized Mean Difference (95% CI)** | ***P* Value** |
|  |  |  |  |
| Average pRNFL  Thickness | Murueta-Goyena/2023 | -0.60 (-1.04 to -0.16) | **0.008** |
|  | Svetozarskiy/2020 | 0.09 (-0.39 to 0.58) | 0.70 |
|  | Kersten/2015 | -0.23 (-1.17 to 0.71) | 0.64 |
|  |  |  |  |
| Temporal pRNFL  Thickness | Murueta-Goyena/2023 | -1.37 (-2.21 to -0.53) | **0.001** |
|  | Svetozarskiy/2020 | -0.11 (-1.50 to 1.28) | 0.87 |
|  | Kersten/2015 | -0.59 (-2.83 to 1.65) | 0.61 |
|  |  |  |  |
| pRNFL, peripapillary retinal nerve fiber layer; OCT, optical coherence tomography; pre-HD, pre-manifest Huntington’s disease; CI, confidence interval.  Boldface values indicate significance of the 95% confidence limit. | | | |

| **Table S9. Quality assessment of the included studies** | | | | | | | | | | | | | |
| --- | --- | --- | --- | --- | --- | --- | --- | --- | --- | --- | --- | --- | --- |
| **Study**  **(First author/Publication year)** | **Selection** | | | | | **Comparability** | | | | **Exposure** | | | |
|  | **Case definition adequacy** | **Representative of cases** | **Selection of controls** | **Definition of controls** | **Subtotal** | **Age** | **Sex** | **Race** | **Subtotal** | **Ascertainment of exposure** | **Same ascertainment** | **Non-response rate** | **Subtotal** |
| Dusek/2023 | * |  |  | * | 2 | * | * |  | 2 | * | * | * | 3 |
| Murueta-Goyena/2023 | * |  |  | * | 2 | * | * |  | 2 | * | * | * | 3 |
| Amini/2022 | * |  |  | * | 2 | * | * |  | 2 | * | * | * | 3 |
| Mazur-Michalek/2022 | * |  |  | * | 2 | * | * |  | 2 | * | * | * | 3 |
| Schmid/2021 | * |  |  | * | 2 |  |  |  | 0 | * | * | * | 3 |
| Di Maio/2020 | * |  |  | * | 2 |  |  |  | 0 | * | * | * | 3 |
| Svetozarskiy/2020 | * |  |  | * | 2 |  |  |  | 0 | * | * | * | 3 |
| Gatto/2018 | * |  |  | * | 2 | * | * | * | 2 | * | * | * | 3 |
| Gulmez Sevim/2018 | * |  |  | * | 2 | * | * |  | 2 | * | * | * | 3 |
| Andrade/2016 | * |  |  | * | 2 | * | * |  | 2 | * | * | * | 3 |
| Kersten/2015 | * |  |  | * | 2 | * | * |  | 2 | * | * | * | 3 |
